# Effect of “Deqi” during the Study of Needling “Wang's Jiaji” Acupoints Treating Spasticity after Stroke

**DOI:** 10.1155/2014/715351

**Published:** 2014-11-12

**Authors:** Huanqin Li, Huilin Liu, Cunzhi Liu, Guangxia Shi, Wei Zhou, Chengmei Zhao, Tao Zhang, Xuefei Wang, Guiling Wang, Yin Zhao, Jingqing Sun, Jing Wang, Linpeng Wang

**Affiliations:** ^1^Acupuncture and Moxibustion Department, Beijing Hospital of Traditional Chinese Medicine Affiliated to Capital Medical University, 23 Meishuguanhou Street, Dongcheng District, Beijing 100010, China; ^2^Acupuncture and Moxibustion Department, Huguosi Hospital of Traditional Chinese Medicine Affiliated to Beijing University of Chinese Medicine, 83 Mianhua Alleyway, Huguosi Street, Xicheng District, Beijing 100035, China; ^3^Traditional Chinese Medicine Department, Fangshan Hospital of Traditional Chinese Medicine, 151 Chengguan South Street, Fangshan District, Beijing 102400, China

## Abstract

*Background.* Acupuncture has been shown to reduce spasticity and prevent the onset of spasticity after stroke. The purpose of this study is to assess the effect of “Deqi” during needling “Wang's Jiaji” acupoints treating spasticity in the early stage of stroke. *Methods*. This study is a multicenter, prospective, randomized, controlled trial. 238 patients with stroke (<21 days) participated and were randomly allocated to the verum-acupuncture (*n* = 121) group or sham-acupuncture group (*n* = 117). The verum-acupuncture group received verum acupuncture required to produce the sense of “Deqi” while the sham-acupuncture group received sham acupuncture without “Deqi.” Patients in both groups followed the same 30 min acupuncture regimen 5 times per week for a period of 4 weeks. Scales of MAS, FMA, ADL, MBI, NIHSS, SS-QOL, and MRS were measured at baseline and at 2, 4, and 12 weeks after intervention. *Results.* Significant differences were observed between two groups. The MRS rating composition has the statistical difference after 4 weeks (*P* = 0.017). The score of MAS, FMA, Barthel, and SSQOL in verum-acupuncture group has increased significantly compared with the sham-acupuncture group after 12 weeks. There was 14% reduction of higher muscle tension in the verum-acupuncture group. *Conclusion.* Acupuncture “Wang's Jiaji” points with sensation of “Deqi” in the early stage may reduce the occurrence and decrease the severity of spasticity after stroke.

## 1. Background

### 1.1. Spasticity after Stroke

Spasticity is explained by a loss of inhibition of reflex activity. Spasticity after stroke is due to damage of advanced brain motor control center, where regulation and inhibition to the low motor center such as spinal cord are interrupted and the initial function of the lower center is released, so the excitability of the motor loop is enhanced. Morbidity of spasticity after stroke is about 65% in the world [1], with about 80%~90% in China [2]. It has the disabling effect on the stroke patients through reduced mobility, which may limit the potential success of rehabilitation [3–5], and affect activities of daily living (ADLs) [6], quality of life (QoL), and work, and add economic and caregiver burdens [3, 7, 8]. Also, the physical limitations associated with spasticity confer risk for falls and consequent fractures [9].

The management of spasticity after stroke should be guided by its potential impact on function and well-being, not only the difficulty with passive muscle stretch or loss of range of motion [10]. Oral medications, nerve blocks, botulinum neurotoxins (BoNT), intrathecal baclofen therapy (ITB), and surgical intervention that try to cure the spasticity have all been tried either separately or in combination but the success rate is not high. More powerful evidence coming from the randomized controlled studies is needed [11]. Study showed that there is compelling evidence that acupuncture (Ac) may have a role in poststroke rehabilitation [12].

### 1.2. Acupuncture

Acupuncture is used in China for more than 3000 years; it has been increasingly integrated into mainstream biomedicine recently [13]. Now it is commonly used in stroke patients [14–16]. It is one of the most important methods to improve the dysfunction after stroke [17]. It has been shown to reduce spasticity [18, 19] and to have the minimal side effect [20]. In particular, early interventions may prevent the onset of spasticity after stroke and slow or limit its progression [21]. Recent systematic reviews suggest that acupuncture may be a useful adjunct to stroke rehabilitation, but stronger conclusions are limited by inappropriate designs, small samples size, and lack of appropriate controls [8, 22, 23]. Most previous studies provide limited objective evidence about the beneficial effects in terms of reduction in spasticity after the acupuncture treatment. A systematic quantification of the ability of acupuncture treatment to reduce spasticity is also lacked. What is more, data shows that any pain stimulation including that produced by acupuncture on limbs may cause the retraction reaction of the flexor and extensor which showed spasticity [24]. The choice of the traditional acupoints used on the limbs to reduce the spasticity is debated [25]. We designed this study, strictly randomized and controlled, with a larger number of cases, to evaluate the efficacy of “Wang's Jiaji” points on the back in spasticity.

*Deqi.* “Deqi” is a composite of unique sensations elicited when acupuncture stimulates. According to the theory of traditional Chinese medicine (TCM), it is regarded that the application of acupuncture through stimulating certain acupoints is to activate “qi” and blood of meridians and collaterals and to regulate the function of internal organs so as to prevent and treat diseases. Therefore, “deqi,” which literally means “the arrival of vital energy,” is a prerequisite for clinical effects and also an important judgment of the exuberance and decline of meridian “qi” and the prognosis of disease [26].

### 1.3. Aims

The aim of our study is to evaluate the efficacy of acupuncture at nontraditional acupoints in poststroke spasticity patients.

## 2. Methods

This study is a prospective, randomized, single blind, controlled clinical trial. We perform this study according to common guidelines for clinical trials (Declaration of Helsinki, International Conference on Harmonisation (ICH)/WHO Good Clinical Practice standards (GCP) including certification by an external audit). The trial protocol has been approved by the Research Ethical Committee of Beijing Hospital of Traditional Chinese Medicine Affiliated to Capital Medical University. This trial was registered with ISRCTN at Current Controlled Trials (ISRCTN84985339).

### 2.1. Participants

All patients treated in the stroke wards in Beijing Hospital of Traditional Chinese Medicine affiliated to Capital Medical University, Huguosi Hospital affiliated to the Beijing University of Chinese Medicine, and Beijing Fangshan Hospital of Traditional Chinese Medicine will be screened according to the following criteria at the inpatient clinic 2 weeks after onset of stroke. The trial is executed from October 2009 to June 2013.

### 2.2. Inclusion Criteria

1. Diagnosis of ischemia stroke, in accordance with the diagnostic criteria for cerebral infarct [27] (including atherosclerotic thrombotic cerebral infarct, cerebral embolism, lacunar infarct).
2. Onset within 21 days (≤21 days).
3. Aged 40–80 years.
4. Scores of NIHSS (National Institute of Health stroke scale) ≥4 and ≤21 points.
5. Scores of GCS (Glasgow coma scale) ≥7 points, without disorder of consciousness.
6. Without severe disability left behind the first stroke.
7. Scores of mRS (modified Rankin scale) ≤1 point.
8. Diagnosed by head CT or MRI.
9. Written and informed consent.

### 2.3. Exclusion Criteria

1. Patients receiving thrombolytic therapy.
2. Limb dystonia caused by other diseases.
3. Subjects tested in other trials in the last 3 months.
4. Combined serious primary heart, liver, kidney, and hematopoietic system diseases and psychiatric patients.
5. Pregnancy or lactating women.
6. Patients with congenitally handicapped patients.

### 2.4. Sample Size

Sample size estimation was based on previous data [28]; the scores of Fugl-Meyer Assessment (FMA) can be improved 36.78 after basic treatment for 6 months. Our previous study [29] indicated that the FMA scores of the verum-acupuncture group can be improved on average of 41.34. According to the comparison of two means, the standard deviation is estimated to be 10.2 points. Based on these assumptions, a sample size of 101 persons in each group is needed to reach a statistical power of 90%. This estimate is based on alpha 0.05. On the assumption that 20% of the participants may fall off during the course of the study, a target of 254 participants has been enrolled. In fact, during the process of study, 263 cases were enrolled, 25 cases fall off, and 238 cases were randomly allocated to the verum-acupuncture group (*n* = 121) and the sham-acupuncture group (*n* = 117).

### 2.5. Randomization and Blinding

Participants are consecutively enrolled and block randomised as they enter the trial at the inpatient clinic. The randomization procedure will be computerized and organized by the Research Center of Clinical Epidemiology affiliated to Peking University. We used block randomization to make the random allocation sequence and prepared predetermined computer-made randomization opaque sealed envelopes. The envelopes are numbered consecutively and were connected into a strain. It is requested that each envelope should be separated from the strain and be opened in sequence only after baseline period when the patient has been registered in the study.

Patients and assessors are blinded about the acupuncture treatment administered. There is only random number not the group number on all the case report form (CRF) tables. Also, all evaluators are not permitted to ask patients about their treatment. Researchers who enroll patients and estimators who collect data in these two hospitals should be trained by the designer about the treatment modalities.

### 2.6. Intervention

The interventions of two groups are operated by the physicians with acupuncture experience for 20 years. There are several approaches of verum/sham acupuncture in both groups including usage of disposable sterile stainless needle (0.32 mm × 40 mm), skin disinfection with 75% alcohol, needles retention for 30 minutes without moxibustion, or electrical stimulation. Patients of both groups received 20 sessions of verum/sham acupuncture in 4 weeks. All the acupuncture practices should be operated by the experienced practitioner after the unified training. Periodic checkup contained the coincidence of the practices taken in each hospital.

In addition to acupuncture, the basic therapies for cerebrovascular disease which are used in all the enrolled patients, including antiplatelet therapy, management of intracranial pressure and blood pressure, neuroprotective agents, treatment of complications, rehabilitation therapy (placement of good posture, routine rehabilitation training, etc.).

#### 2.6.1. Verum Acupuncture

“Wang's Jiaji” points selected from Jiaji (EX-B2) are the necessary points used in acupuncture group, including the points located 0.3 cun lateral to the lower border of the 2nd, 4th, 6th, 8th, 10th, and 12th thoracic vertebra, and the 2nd and 4th lumbar vertebra. The patients are required to keep lateral position, hemiplegia limbs upwards. Piercing vertically, needles are inserted 10–25 mm in depth and manually manipulated by lifting, thrusting, and rotating methods with uniform reinforcing-reducing techniques to produce the sense known as “deqi.”

#### 2.6.2. Sham Acupuncture

The points used in the sham acupuncture group located 0.1 cun lateral to the lower border of the 2nd, 4th, 6th, 8th, 10th, and 12th thoracic vertebra and the 2nd and 4th lumber vertebra. The patients are required to keep lateral position, hemiplegia limbs upwards. Piercing vertically, needles are inserted 5 mm in depth and remained for 30 minutes without moxibustion or electrical stimulation, with no needling sensation.

#### 2.6.3. Procedure

**Figure 1:**
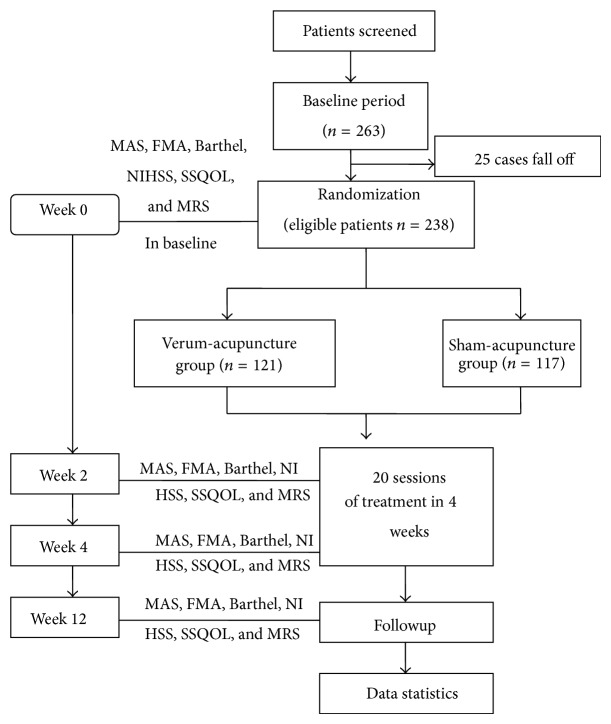
Technology roadmap.

The intervention procedure is as follows. See Figure 1.

### 2.7. Outcome Measures

The efficacy of acupuncture for spasticity after stroke is assessed by the following primary and secondary outcome measures; they will be assessed at baseline and at weeks 2, 4, and 12.

The main outcome measures are as follows.

1. The modified Ashworth scale (MAS) was used to assess the muscle tension.
2. The motor function was assessed by the Fugl-Meyer Assessment (FMA), including the upper and lower limbs.

The secondary outcome measures are as follows.

1. The life ability was assessed by Activites of Daily Living (ADL) Scale with the modified Barthel index (MBI).
2. The neural function defect was assessed by the revised National Institute of Health Stroke Scale (NIHSS).
3. The quality of life was assessed by stroke specialization quality of life scale (SS-QOL).
4. The independent life ability was assessed by the modified Rankin scale (MRS).

## 3. Statistical Analysis

The statistical analysis was performed by the Epidemiological Research Center of the Third Affiliated Hospital of Beijing University. Mean is used to describe the central tendency of continuous variables; the standard deviation is used to describe the discrete case. Kolmogorov-Smirnov method is used to test the normality of continuous variables. The data in line with the normal distribution will be analyzed using *t*-test to compare the differences between therapy group and control group. Nonparametric data will be analyzed using the Wilcoxon test to compare the difference between the two groups. If the outcome variables are two-way 2 × 2 contingency tables, chi-square tests or Fisher exact probability tests will be performed. In order to compare the effect of two groups before and after treatment, Mcnemer paired chi-square test is used to compare the difference before and after treatment in each group.

Statistical analyses will be conducted using SPSS version 18.0. The *P* value cutoff for statistical significance is defined as *P* < 0.05, and all statistical tests are two-tailed.

## 4. Results

From March 2009 to March 2013, 263 cases were included, 238 cases were completed, 121 cases belong to the treatment group, and 117 cases belong to the control group. 25 cases fall off; the rate of falls off was 9.50%. (During the process of the study, slow progress occurred due to the complicated inclusion of more cases, and we screened the Beijing Fangshan Hospital of Traditional Chinese Medicine as the third center to include the subjects. Of course, the researchers involved in the study received the same training.)

The gender and age of patients between the treatment group and control group has no statistical difference; it is comparable of the onset time between two groups. The scores of MAS, FMA, Barthel index, NIHSS, SSQOL, and MRS were not statistical differences between the treatment group and control group (Tables 1, 2, 3, 4, and 5).

The MRS rating composition between the verum-acupuncture group and sham-acupuncture group has the statistical difference after 4 weeks, but there is no statistical difference at the time of 2 weeks and 12 weeks after treatment (Tables 6, 9, and 13).

The score of MAS, FMA, Barthel, and SSQOL has increased significantly compared with that in control group after 12 weeks. But the score of NIHSS has no statistical difference at the time of 2, 4, and 12 weeks after treatment (Tables 7, 10, and 14).

The differences between groups about the change value of MAS, FMA, Barthel, and SSQOL were statistically significant at 4 and 12 weeks after treatment compared to baseline. The change value of NIHSS score compared to the baseline had no statistical difference (Tables 8, 11, and 15).

Furthermore, the MAS of 37 cases was ≥3, 84 cases <3; the rate of higher muscle tension was 44%, which was below the 83% of the control group after 4 weeks of treatment. After 12 weeks of treatment, the MAS of 28 cases was ≥3, 93 cases <3 in the verum-acupuncture group; the rate of higher muscle tension was 30%, which was below the 60% of the control group after 12 weeks of treatment. There was 14% reduction of higher muscle tension in the verum-acupuncture group (Tables 12 and 16).

## 5. Discussion

Spasticity after stroke especially of the affected limbs is a common complication which seriously impacts the quality of life of stroke patients, and it is also an important factor affecting the movement of stroke patients. The main purpose of this study is to define the acupuncture in the early stage of poststroke which may effectively relieve specificity or not, including reducing the probability of occurrence and decreasing severity of spasticity, on this basis, whether it affects damage and disability of patients or not, including the NIHSS, motor function, and quality of life.

Acupuncture may decrease the increased spinal motor neuron excitability in paretic limbs of stroke patients [30]. The application of “Wang's Jiaji” points in acute stage of stroke patients may prevent the onset of spasticity, reduce the degree of spasticity [31], and improve the QOL of stroke patients in our preliminary study. And it is effective in spasticity superior to the conventional acupuncture in combination with rehabilitation training and the simple rehabilitation training [32]. Also, the effect is demonstrated to improve the hypertonia of the spasticity rat [33], but more advanced design is needed. Also, we had not found the similar study about Jiaji (EX-B2). The emphasis of this trial is strictly concealed randomization and the successful blinding of patients. All the physicians applying acupuncture treatment in this trial have more than 10 years of clinical experience of acupuncture.

The occurrence of spasticity is highly variable and may occur in the short-, medium-, or long-term poststroke period [34]; these variations in the time of onset of poststroke undermine efforts at measuring spasticity prevalence. Despite the fact that there is no clear consensus regarding the number of patients who develop spasticity after stroke [34], spasticity had emerged in the early stage of poststroke (5.4 days to 2 weeks) in recent study [34, 35]. Because of the high onset rate of spasticity, especially in the early stage poststroke, we chose the acute stroke patients (<21 days) as included cases.

The study found that the scores of FMA of 12 weeks after treatment in the verum-acupuncture group were higher than the sham-acupuncture control group. The rate of higher muscle tension (MAS ≥ 3) in verum-acupuncture group decreased obviously after 4 and 12 weeks after treatment, especially after 12 weeks compared to sham-acupuncture group. Modified Ashworth scale (MAS) is now commonly used to assess the muscle tension and the Fugl-Meyer Assessment (FMA) is used to assess the motor function, including the upper and lower limbs. Therefore, we can draw the conclusion that acupunctures “Wang's Jiaji” points in the stage of stroke may reduce spasticity and decrease the severity of spasticity; it can effectively promote the movement of patients with stroke function improvement.

The study also found that the score of MBI and SSQOL at time of 12 weeks after treatment in the verum-acupuncture group was better than the sham-acupuncture control group. According to that, we concluded that acupuncture “Wang's Jiaji” points early may improve the life ability and quality of life.

What is more, the change in value of MAS, FMA, MBI, and SSQOL between groups was found at 4 weeks after treatment compared to the baseline, and it was more obvious at 12 weeks. The effect of acupuncture “Wang's Jiaji” points early in the stroke patients appeared at the time of 4 weeks after treatment. But it is strange that the score of NIHSS was not found to be improved during the study at 2, 4, and 12 weeks after treatment, even compared to the baseline.

The spasticity degree of limbs is an important factor which affects the motor function. Decreasing of spasticity can effetely improve the motor function. The recovery of limbs function of stroke patients needs long process, it is found that lower incidence and lighter degree of spasticity in verum-acupuncture group make the motor function benefit by interview 12 weeks after treatment. The score of NIHSS is used to reflect the overall level of stroke in patients with neurologic impairment, but there is only 2 items regarding the motor function of limbs. The improvement of motor function is difficult to be reflected by the score of NIHSS. In our study, the beneficial effects to score of NIHSS was not found because the improvement of limbs spasticity.

“Wang's Jiaji” points come from the experience of Dr. Wangle ting who worked as an acupuncture doctor for 60 years. He believes that the position of “Wang's Jiaji” points is easier stimulated to “deqi” when acupuncture is compared to traditional Jiaji (EX-B2). We also believe that “deqi” during acupuncture at “Wang's Jiaji” points was helpful in the effective results of this study.

According to the view of modern neurophysiology, the posterior and anterior rami of spinal nerve and the sympathetic trunk are distributed in the region of “Wang's Jiaji” acupoints, with each of the posterior rami of spinal nerve connected with the adjacent 1-2 posterior ramus nerve upper and lower by fiber. It is effective that needling Wang's Jiaji acupoints to prevent and decrease the occurrence of spasticity by affecting the interactions of spinal cord and motor neurons, adjusting the spinal stretch reflex and balancing the function of motion system, which may adjust the state of the limb muscle tension. The study by Fink et al. [36] indicates that needle acupuncture may not be helpful to patients with chronic poststroke spasticity. But there was neurophysiologic evidence for specific acupuncture effects on a spinal (segmental) level involving nociceptive reflex mechanisms. Therefore, the purpose of our trial is to discuss the efficacy of needling unconventional acupoints such as Wang's Jiaji points on preventing and decreasing spasticity after stroke. The results of this trial will be helpful to supply the evidence.

The position of “Wang's Jiaji” acupoints is prone to produce “deqi”; in particular, the technique and depth of acupuncture stimulation in the verum group decided the produce of “deqi” better than the sham group. So we think that the effect of “deqi” during the acupuncture treatment is very important which will affect the curative effect about improvement of limb muscle tension which is a benefit for the stroke patients.

Our study has some limitations. Firstly, MAS is subjective and the Barthel index score or NIHSS cannot indicate the spasticity directly. We will add other objective measures which will be more appropriate to evaluate spasticity such as electrophysiology or biomechanics in future study. Secondly, we consider that a longer period of followup could be necessary to investigate the optimum timing for such an acupuncture treatment and to assess the value of repeated courses of acupuncture for patients experiencing spasticity after stroke.

## 6. Conclusion

It is helpful to reduce the occurrence and decrease the severity of spasticity after stroke by acupuncture “Wang's Jiaji” points in early stroke stage, especially for the patients whose occurrence of stroke is within 21 days, with limb muscle strength less than or equal to 3 evaluated by MAS.

## Figures and Tables

**Table 1:** Contrast of gender between treatment group and control group before treatment.

	Treatment group (*n* = 121)		Control group (*n* = 117)		Chi-square value	*P*
	*n*	%	*n*	%		
Gender						
Male	78	64.50%	81	69.2%		
Female	43	35.50%	36	30.8%	0.61	0.435

**Table 2:** Contrast of age between treatment group and control group before treatment.

	Treatment group		Control group		*T*	*P*
	Mean	SD	Mean	SD		
Age (Y)	63.2	10.485	64.21	10.193	−0.751	0.454

**Table 3:** Contrast of time onset of stroke between treatment group and control group before treatment.

	Treatment group		Control group		*Z*	*P*
	Mean	SD	Mean	SD		
Time onset (day)	10.93	6.973	12.19	7.452	1.412	0.158

**Table 4:** Contrast of the rate of MRS before treatment.

	Rate of MRS						Chi-square value	*P*
	0	1	2	3	4	5		
Treatment group	0	1	1	24	81	14	6.637	0.183
Control group	2	0	2	35	66	2		

**Table 5:** The scores of MAS, FMA, Barthel, NIHSS, and SSQOL between treatment group and control group before treatment.

	Treatment group		Control group		*T*	*P*
	Mean	SD	Mean	SD		
MAS_	12.47	7.468	13.01	6.137	−0.605	0.545
FMA	30.32	21.573	31.52	18.962	−0.455	0.650
Barthel	33.72	15.699	36.98	16.125	−1.582	0.115
NIHSS	11.13	4.649	10.62	4.386	0.882	0.379
SSQOL	102.74	31.147	106.09	35.762	−0.769	0.442

**Table 6:** Contrast of the rate of MRS after 2 weeks of treatment.

	Rate of MRS						Chi-square value	*P*
	0	1	2	3	4	5		
Treatment group	0	10	10	26	68	7	6.215	0.250
Control group	0	14	17	29	49	8		

**Table 7:** The scores of MAS, FMA, Barthel, NIHSS, and SSQOL between treatment group and control group after 2 weeks of treatment.

	Treatment group		Control group		*T*	*P*
	Mean	SD	Mean	SD		
MAS_	18.05	10.463	17.73	9.014	0.255	0.799
FMA	45.80	25.439	44.31	18.062	0.565	0.573
Barthel	47.44	21.116	47.03	18.824	0.158	0.874
NIHSS	9.01	7.808	9.21	8.630	−0.530	0.597
SSQOL	128.19	38.191	123.65	35.605	0.948	0.344

**Table 8:** The change in value of scores of MAS, FMA, Barthel, NIHSS, and SSQOL between two groups after 2 weeks of treatment compared to the baseline.

	Treatment group		Control group		*T*	*P*
	Mean	SD	mean	SD		
MAS_	5.579	9.291	4.718	6.438	0.828	0.409
FMA	15.479	20.408	12.786	12.432	1.224	0.222
Barthel	13.719	17.566	10.026	12.314	1.867	0.063
NIHSS	−2.066	−4.487	1.915	4.228	−0.268	0.789
SSQOL	25.446	37.96	18.552	15.760	2.178	0.07

**Table 9:** Contrast of MRS rating between treatment group and control group after 4 weeks of treatment.

	MRS rating						Chi-square value	*P*
	0	1	2	3	4	5		
Treatment group	0	18	30	44	27	2	11.437	0.017
Control group	0	14	27	33	41	2		

**Table 10:** The scores of MAS, FMA, Barthel, NIHSS, and SSQOL between treatment group and control group after 4 weeks of treatment.

	Treatment group		Control group		*T*	*P*
	Mean	SD	Mean	SD		
MAS_	23.98	10.835	21.78	9.461	1.664	0.097
FMA	56.45	24.05	51.95	21.783	1.450	0.148
Barthel	55.42	21.70	55.21	20.797	1.525	0.129
NIHSS	6.78	3.203	7.34	3.138	−1.374	0.171
SSQOL	145.29	32.978	136.60	42.97	1.771	0.078

**Table 11:** The change in value of scores of MAS, FMA, Barthel, NIHSS, and SSQOL between two groups after 4 weeks of treatment compared to the baseline.

	Treatment group		Control group		*T*	*P*
	Mean	SD	Mean	SD		
MAS_	11.504	10.453	8.769	7.824	2.279	0.024
FMA	26.132	23.718	20.615	14.582	2.153	0.032
Barthel	25.703	20.609	18.231	17.710	2.995	0.003
NIHSS	−4.298	4.79	−3.778	4.159	−0.893	0.373
SSQOL	42.546	37.306	30.513	28.439	2.791	0.006

**Table 12:** The distribution of higher muscle tension patients (Ashworth scores ≥3) in the treatment group and control group after 4 weeks of treatment.

	Ashworth score		Chi-square value	*P*
	≥3	<3		
Treatment group	37	84	5.481	0.019
Control group	53	64		

**Table 13:** Contrast of MRS rating between treatment group and control group after 12 weeks of treatment.

	MRS rating						Chi-square value	*P*
	0	1	2	3	4	5		
Treatment group	2	23	44	39	11	2	7.621	0.151
Control group	0	25	28	43	19	2		

**Table 14:** The scores of MAS, FMA, Barthel, NIHSS, and SSQOL between treatment group and control group after 12 weeks of treatment.

	Treatment group		Control group		*T*	*P*
	Mean	SD	Mean	SD		
MAS_	30.78	9.448	25.91	10.525	3.753	0.000
FMA	67.90	21.695	56.42	23.825	3.89	0.000
Barthel	71.61	21.911	61.62	21.059	3.583	0.000
NIHSS	5.36	3.284	5.62	3.365	−0.604	0.546
SSQOL	169.96	38.694	146.72	44.565	4.30	0.000

**Table 15:** The change in value of scores of MAS, FMA, Barthel, NIHSS, and SSQOL between two groups after 12 weeks of treatment compared to the baseline.

	Treatment group		Control group		*T*	*P*
	Mean	SD	Mean	SD		
MAS_	18.306	9.073	12.906	9.876	4.395	0.000
FMA	37.759	22.376	24.897	19.737	4.631	0.000
Barthel	37.893	20.522	24.641	18.761	5.194	0.000
NIHSS	−5.711	5.021	−5.496	4.408	−0.351	0.726
SSQOL	67.215	39.603	40.632	33.334	5.593	0.000

**Table 16:** The distribution of higher muscle tension patients (Ashworth scores ≥3) in the treatment group and control group after 4 weeks of treatment.

	Ashworth score		Chi-square value	*P*
	≥3	<3		
Treatment group	28	93	5.90	0.015
Control group	44	73		
